# Supplementary material for: Resolving the full spectrum of human genome variation using Linked-Reads
Source: Genome Res. 2019 Apr;29(4):635–45. doi: 10.1101/gr.234443.118 (PMC6442396; doi:10.1101/gr.234443.118)
Supplement: Supplemental Material [file supp_gr.234443.118_Supplemental_longranger-2.2.zip › longranger-2.2.tar.bz2!/lr2.2-public/lib/stan/cmdstan/stan/doc/api/html/init__windowed__adapt_8hpp_source.html]

Stan: src/stan/common/init\_windowed\_adapt.hpp Source File


|  |  |
| --- | --- |
|  | Stan  2.5.0  probability, sampling & optimization |


- Main Page
- Related Pages
- Namespaces
- Classes
- Files

- File List
- File Members

All Classes Namespaces Files Functions Variables Typedefs Enumerations Enumerator Friends Macros Pages

- src
- stan
- common

init\_windowed\_adapt.hpp

Go to the documentation of this file.

1 #ifndef STAN\_\_COMMON\_\_INIT\_WINDOWED\_ADAPT\_HPP

2 #define STAN\_\_COMMON\_\_INIT\_WINDOWED\_ADAPT\_HPP

3

4 #include <stan/mcmc/base\_mcmc.hpp>

5 #include <stan/gm/arguments/categorical\_argument.hpp>

6 #include <stan/math/matrix/Eigen.hpp>

7 #include <stan/common/init\_adapt.hpp>

8

9 namespace stan {

10  namespace common {

11

12  template<class Sampler>

13  bool init\_windowed\_adapt(stan::mcmc::base\_mcmc\* sampler,

14  stan::gm::categorical\_argument\* adapt,

15  unsigned int num\_warmup,

16  const Eigen::VectorXd& cont\_params) {

17

18  init\_adapt<Sampler>(sampler, adapt, cont\_params);

19

20  unsigned int init\_buffer

21  = dynamic\_cast<stan::gm::u\_int\_argument\*>(adapt->arg("init\_buffer"))->value();

22  unsigned int term\_buffer

23  = dynamic\_cast<stan::gm::u\_int\_argument\*>(adapt->arg("term\_buffer"))->value();

24  unsigned int window = dynamic\_cast<stan::gm::u\_int\_argument\*>(adapt->arg("window"))->value();

25

26  dynamic\_cast<Sampler\*>(sampler)->set\_window\_params(num\_warmup, init\_buffer,

27  term\_buffer, window, &std::cout);

28

29  return true;

30

31  }

32

33  }

34 }

35

36 #endif

base\_mcmc.hpp

categorical\_argument.hpp

stan::gm::singleton\_argument

**Definition:** singleton\_argument.hpp:44

stan::gm::categorical\_argument

**Definition:** categorical\_argument.hpp:11

Eigen.hpp

init\_adapt.hpp

stan::common::init\_windowed\_adapt

bool init\_windowed\_adapt(stan::mcmc::base\_mcmc \*sampler, stan::gm::categorical\_argument \*adapt, unsigned int num\_warmup, const Eigen::VectorXd &cont\_params)

**Definition:** init\_windowed\_adapt.hpp:13

stan::mcmc::base\_mcmc

**Definition:** base\_mcmc.hpp:13


---

[ Stan Home Page ]

*© 2011–2014,
Stan Development Team.*
